# Exploring the associations between systemic inflammation, obesity and healthy days: a health related quality of life (HRQOL) analysis of NHANES 2005–2008

**DOI:** 10.1186/s40608-018-0196-2

**Published:** 2018-08-06

**Authors:** Jeffrey Wilkins, Palash Ghosh, Juan Vivar, Bibhas Chakraborty, Sujoy Ghosh

**Affiliations:** 1 Biomedical Biotechnology Research Institute, North Carolina Central University, 1801 Fayetteville Street, Durham, NC 27707 USA; 2 Centre for Quantitative Medicine, Duke-NUS Medical School, 8 College Road, Singapore, 169857 Singapore; 3 Center for Tobacco Products, Food and Drug Administration, 10903 New Hampshire Avenue, Silver Spring, MD 20993 USA; 4 Program in Cardiovascular & Metabolic Disorders & Centre for Computational Biology, Duke-NUS Medical School, 8 College Road, Singapore, 169857 Singapore

**Keywords:** Obesity, Inflammation, Healthy days, Health-related quality of life, Mediation

## Abstract

**Background:**

Obesity is positively associated with low-level chronic inflammation, and negatively associated with several indices of health-related quality of life (HRQOL). It is however not clear if obesity-associated inflammation is partly responsible for the observed negative associations between obesity and HRQOL, and also whether systemic inflammation independently affects HRQOL. We conducted an exploratory analysis to investigate the relationships between obesity, systemic inflammation and indices of HRQOL, using NHANES survey data.

**Methods:**

Data for the variables of interest were available for 6325 adults (aged 20–75 years, BMI > 18.5 kg/m^2^). Demographic, body mass index (BMI), C-reactive protein (CRP), inflammatory disease status, medication use, smoking, and HRQOL data were obtained from NHANES (2005–2008) and analyzed using sampling-weighted generalized linear models. Data was subjected to multiple imputation in order to mitigate information loss from survey non-response. Both main effects and interaction effects were analyzed to evaluate possible mediation or moderation effects. Model robustness was ascertained via sensitivity analysis. Averaged results from the imputed datasets were reported in as odds ratios (OR) and confidence intervals (CI).

**Results:**

Obesity was positively associated with poor physical healthy days (OR: 1.59, 95% CI: 1.15–2.21) in unadjusted models. ‘Elevated’ and ‘clinically raised’ levels of the inflammation marker CRP were also positively associated with poor physical healthy days (OR = 1.61, 95% CI: 1.23–2.12, and OR = 2.45, 95% CI: 1.84–3.26, respectively); additionally, ‘clinically raised’ CRP was positively associated with mental unhealthy days (OR = 1.66, 95% CI: 1.26–2.19). The association between obesity and physical HRQOL was rendered non-significant in models including CRP. Association between ‘elevated’ and ‘clinically raised’ CRP and physical unhealthy days remained significant even after adjustment for obesity or inflammation-modulating covariates (OR = 1.36, 95% CI: 1.02–1.82, and OR = 1.75, 95% CI: 1.21–2.54, respectively).

**Conclusions:**

Systemic inflammation appears to mediate the association between obesity and physical unhealthy days. Clinically raised inflammation is an independent determinant of physical and mental unhealthy days. Importantly, elevated (but sub-clinical) inflammation is also negatively associated with physical healthy days, and may warrant more attention from a population health perspective than currently appreciated.

**Electronic supplementary material:**

The online version of this article (10.1186/s40608-018-0196-2) contains supplementary material, which is available to authorized users.

## Background

Obesity poses one of the most significant public health challenges of the developed and developing nations today. The fundamental process underlying obesity is an energy imbalance between calories consumed and calories expended, resulting in a net positive energy balance. Obesity has accelerated globally due to an increased intake of energy-dense food high in fat and refined carbohydrates, reduced physical activity associated with an increasingly sedentary lifestyle, altered modes of transportation, control of ambient temperature and increasing urbanization [1, 2]. Worldwide obesity rates have nearly tripled between 1975 and 2016, with more than 650 million adults obese worldwide. In addition to weight-related pathologies, obesity is also a gateway to other chronic disorders including type 2 diabetes, cardiovascular disease, musculoskeletal disorders, and specific cancers [3–5]. Regardless of origin, higher levels of obesity are associated with higher relative mortality risk compared to healthy weight [6]. Consequently, obesity and obesity-associated health problems lead to a significant economic impact involving direct and indirect medical costs [7–9].

Obesity is typically associated with a chronic state of systemic low-grade inflammation [10]. Rapid adipose tissue expansion due to overnutrition results in a hypoxic internal core which, along with endoplasmic reticulum and oxidative stress, orchestrates a pro-inflammatory response through the release of various cytokines into systemic circulation [11]. Adipose tissue depots also undergo significant immune cell infiltration further contributing to a sustained inflammatory tone [12, 13]. Although biological studies have strongly implicated a causal role of obesity in promoting inflammation [14–18], Mendelian randomization experiments employing genetic variants in the inflammation biomarker C-reactive protein (CRP) [19] and obesity-associated FTO gene [20–22] have formally established the direction of causality from increased adiposity to elevated systemic inflammation [23]. The same technique has also been recently used to infer a causal role of obesity in promoting inflammatory skin diseases [24]. Obesity-associated chronic inflammation, in turn, has been causally associated with several metabolic complications including insulin resistance, endothelial dysfunction, and type 2 diabetes [5, 25–29].

From a population health perspective, effective interventions and optimized predictions for future health-care costs require a better quantitative understanding of chronic conditions such as inflammation and obesity and their relationship to indices of public health. One index that captures the population level effects of chronic conditions is the health related quality of life (HRQOL) [30]. HRQOL, a self-reported measure of physical and mental functioning and well-being, is increasingly used to assess the effects of chronic illness, treatments, and short- and long-term disabilities.

Previous studies have generally demonstrated a negative correlation between excess adiposity and various dimensions of HRQOL in different populations [31–37]. Much less is known, however, on the effects of chronic inflammation on HRQOL, and whether inflammation mediates some of the association between obesity and HRQOL. Most of the earlier studies investigating inflammation and HRQOL have either focused on small cohorts targeting specific inflammatory disorders [38–40], or interrogated inflammation and HRQOL as separate endpoints. Recently however, the role of systemic inflammation as a co-factor in the disease to HRQOL relationship is beginning to be examined [41, 42]. Systemic inflammation is often measured via the chronic inflammatory biomarker, C-reactive protein (CRP) [43, 44], which is a commonly used predictor in studies of inflammatory disease, and considered an excellent biomarker of baseline and progressive inflammation in chronic conditions [45]. CRP levels in the range consistent with infection or inflammation (> 1 mg/dl) are more common among obese subjects than in non-obese subjects [46]. Additionally, reliable associations between blood levels of the inflammation biomarker CRP and a variety of HRQOL outcomes have also been reported [47, 48].

The temporal precedence of obesity over systemic inflammation, and the reported causal connections between obesity-associated inflammation and secondary disorders [25], raises the question of whether obesity-associated chronic inflammation may also play a similar role in the observed association between obesity and HRQOL. More specifically, such questions may be explored in the mediator-variable framework of Barron and Kenny [49, 50] whereby an antecedent variable (e.g. obesity) may affect a mediating variable (e.g. inflammation), which would then affect an outcome variable (e.g. HRQOL). While acknowledging the potential biases of a cross-sectional mediation analysis, the strong prior biological evidence causally linking obesity to inflammation, and inflammation to obesity-associated disorders led us to explore a similar scenario for obesity and HRQOL. This line of questioning has important implications for prevention and treatment research where interventions may be designed to alter the outcome of interest by controlling the mediating variables, especially where the primary variable is difficult to control. Thus, if chronic inflammation does indeed mediate the association between obesity and poorer HRQOL, then controlling for such inflammation via lifestyle and pharmacologic interventions may provide a path forward for improving the HRQOL index for the obese population. This is particularly relevant, given the current paucity of effective treatments for obesity [51].

## Methods

### Study design and participants

Study data was downloaded from the National Health and Nutrition Examination Survey (NHANES) collection (years 2005 through 2008, http://www.cdc.gov/nchs/nhanes/nhanes_questionnaires.htm). NHANES is conducted by the Center for Disease Control’s National Center for Health Statistics (CDC-NCHS) to assess the health and nutritional status of a representative civilian, non-institutionalized US population using a multistage, stratified, clustered probability design [52]. Data for the variables of interest were available for a total of 6325 adults (aged 20–75 years, BMI > 18.5 kg/m^2^) and included missing values. Data for subjects 18–20 years of age were excluded due to the large excess of missing values in this group and to prevent complications from differential growth patterns in childhood and adolescence where the usual BMI categories do not apply [53]. There were no missing values for age, sex and race variables. For the other variables, the extent of missing value ranged from 0.01% (presence of heart disease) to ~ 53% (smoking). BMI and CRP had missing values for ~ 6 and 10% of the observations respectively, whereas both physical and mental healthy days had missing values of ~ 12%. Data collected included demographic information, health-related questionnaire, and laboratory data on C-reactive protein (CRP, a marker of systemic inflammation). Data for confounding factors that can influence inflammation status and HRQOL outcomes such as relevant medical conditions (asthma, arthritis, heart disease, and cancer), anti-inflammatory/analgesic medication, and smoking were also downloaded for statistical modeling. To account for the complexity of survey design including oversampling, survey non-response, or post-stratification issues, NHANES assigns sample ‘weights’ were also downloaded (additional details available from https://www.cdc.gov/nchs/tutorials/nhanes/SurveyDesign/Weighting/OverviewKey.htm, and in Additional file 1: Text 1).

### Health related quality of life (HRQOL) measures

Quality of life was assessed by using a subset of the CDC HRQOL-4 questionnaire that was developed to assess physical and mental health in the general U.S population [54, 55]. The HRQOL-4 questionnaire **(**Additional file 1: Text 2) uses self-reported measures of healthy and unhealthy days as indicators of HRQOL, and have undergone cognitive testing and criterion validity with the Short-Form 36, content and construct validity, predictive validity, internal consistency, test-retest reliability, and measurement invariance in persons with and without disability [56, 57]. The core Healthy Days consists of four questions focusing on the participant’s general health status (Question-1), number of physical unhealthy days in the 30 days preceding the survey (Question-2), number of mental unhealthy days in the 30 days preceding the survey (Question- 3), and number of days with activity limitations in the 30 days preceding the survey (Question-4). Question-1 is a predictor of mortality and chronic disease conditions [58], questions − 2 and − 3 assess recent physical symptoms and mental or emotional distress, respectively, and question- 4 measures perceived disability and lost productivity [55]. Only responses to survey Questions-2 and -3 were used in the current study since the focus of the analysis was to determine effects specifically on physical and mental health. Throughout this analysis, an *increase* in the number of physical/mental unhealthy days has been used to indicate poorer health outcomes.

### Coding of variables

Participants were divided into 3 categories by age (20–44 yrs., 45–65 yrs., > 65 yrs.) and 5 categories by race/ethnicity as Mexican-American (1), Other Hispanic (2), Non-Hispanic White (3), Non-Hispanic Black (4), and Other (5). Obesity was measured by body mass index (BMI) based on self-reported weight in kilograms divided by measured height in meters-square. Respondents were broadly categorized into 3 BMI groups: normal weight (BMI 18.5–24.9), overweight (BMI 25–29.9), and obese (BMI > 30). Systemic inflammation was measured via blood CRP levels (mg/dl) and grouped into 3 classes according to Visser et al. [44] – non-elevated CRP (< 0.22 mg/dl), elevated CRP (≥0.22 and < 1.0 mg/dl) and clinically raised CRP (≥1.0 mg/dl), respectively. Each medical condition, including asthma (MCQ010), arthritis (MCQ160A), cancer (MCQ220), or any heart disease (a composite variable derived from a positive diagnosis of any one of congestive heart failure (MCQ160B), coronary heart disease (MCQ160C), or heart attack (MCQ160E)) were dichotomized into ‘0’ and ‘1’ categories where ‘1’ indicates a positive response to the question of whether there ever was a diagnosis of the relevant condition by a doctor or healthcare professional. Smoking status was also dichotomized, with ‘1’ assigned to individuals who are current smokers. The use of common analgesic and anti-inflammatory medications (aspirin, acetaminophen, ibuprofen and naproxen) was extracted from the RXDDRUG field of NHANES data, which records the generic name of the drug. Subjects associated with any one of the 4 drugs listed above were marked with ‘1’ and ‘0’ otherwise. Although acetaminophen is more widely prescribed as an analgesic and antipyretic rather than anti-inflammatory drug, previous studies have reported effectiveness of acetaminophen against lower grade inflammation [59] and acetaminophen overdose has been associated with reductions in circulating CRP levels [60]. Based on these findings, and the close association between inflammation and physical pain, we included the use of acetaminophen in the analysis. For logistic regression analysis each outcome variable (HRQOL measures) was dichotomized into ≤15 or > 15 days of poor physical (HSQ470) or mental health (HSQ480), with > 15 unhealthy days denoted by 1, and 0 otherwise. The rationale for dichotomization was based on earlier reports which considered a report of > 14 days of poor physical or mental health as representing a state of ‘frequent distress’ [61, 62].

### Statistical analysis

All statistical analysis was conducted using SAS, version 9.1 (SAS Institute Inc., Cary, NC, USA) or R (version × 64 3.2.3). Data was analyzed using sampling weighted generalized linear models. Both unadjusted and adjusted models linking BMI and CRP to the outcome variables (HSQ470 and HSQ480) were constructed, with adjustments for possible inflammation-regulating medical conditions (asthma, arthritis, heart disease, and cancer), use of over the counter anti-inflammatory/pain medications (acetaminophen, aspirin, ibuprofen and naproxen), and current cigarette smoking status. As several of the surveyed variables contained missing values, any attempt to analyze only complete cases severely reduced the total number of observations, leading to increased risk of biased estimates. To mitigate this problem, we used multiple imputation [63] to estimate probable value ranges for incomplete observations. The original coding for the missing values included bona-fide missing values, plus other types of non-response such as “don’t know” (1517 total instances), and “refused to answer” (2 total instances). We converted all missing and non-response cases into missing values, as recommended in the NHANES analytical guidelines [64]. Multiple imputation generates more than one set of replacements for the missing values based on plausible models for data thereby yielding multiple completed datasets for analysis [65]. Five imputed datasets were generated according to multiple imputation procedures described by Rubin [63]. Each of these “completed” datasets were individually analyzed using sampling weighted generalized linear models (GLM), via the *survey* package in R [66]. For each of the imputed datasets, *m* = 1…5, we obtained the estimate of regression coefficient as *β*_*m*_ along with the standard error *s*_*m*_. The overall estimate was obtained by averaging the individual estimates from the imputed datasets as

$$ \widehat{\beta}=\frac{1}{M}\sum \limits_{m=1}^M{\widehat{\beta}}_m $$

The estimated variance for $\widehat{\beta}$ is given by

$$ V\left(\hat{\beta}\right)=W+\left(1+\frac{1}{M}\right)B, $$

$$ \mathrm{where}\kern0.48em W=\frac{1}{M}{\sum}_{m=1}^M{s}_m^2,\kern0.36em \mathrm{and}\kern0.36em B=\frac{1}{M-1}{\sum}_{m=1}^M{\left(\hat{\beta_m}-\hat{\beta}\right)}^2 $$

Due to the difficulties in combining and interpreting the combined *p*-values arising from the above analysis, we have chosen to report results in terms of the estimated odds ratio (${e}^{\widehat{\beta}}$) and their 95% confidence interval for all analysis. The odds ratios (OR) were obtained by exponentiation of the average regression coefficient, $\widehat{\beta}$.

**Fig. 1:**
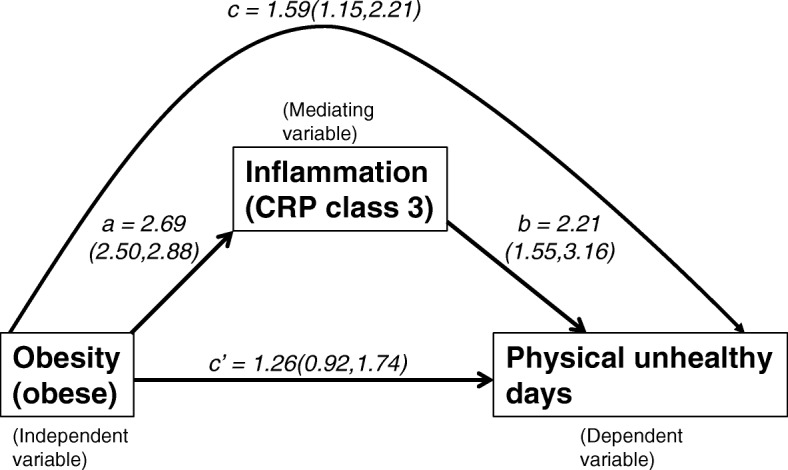
Effect of adjusting for inflammation on the estimate of obesity-physical unhealthy days association in NHANES subjects. The ‘direct effect’ estimates the odds ratio (and confidence intervals) for the association of adult obese subjects to the number of physical unhealthy days, a measure of HRQOL. The ‘indirect effect’ estimates the same association following the inclusion of clinically raised systemic inflammation (measured as C-reactive protein) as a possible mediator. The odds ratios (and confidence intervals) for direct associations of obesity to inflammation and of inflammation to physical unhealthy days are also shown

The mediation analyses that are presented in Fig. 1, consisted of the following steps, as suggested by MacKinnon et al. [67]. The main association between the independent variable (obesity) and dependent variable (physical unhealthy days) were examined as per Eq. 1 (*c*-coefficient in Fig. 1). Next, we estimated the association of the mediating variable (inflammation, CRP levels) to the dependent variable in the presence of obesity (Eq. 2, *b*-coefficient in Fig. 1). Finally, we investigated the association of the mediating variable to the independent variable (Eq. 3, *a*-coefficient in Fig. 1).

1 $$ \mathrm{Y}={\mathrm{i}}_1+\mathrm{cX}+{\mathrm{e}}_1 $$

2 $$ \mathrm{Y}={\mathrm{i}}_2+{\mathrm{c}}^{\hbox{'}}\mathrm{X}+\mathrm{bM}+{\mathrm{e}}_2 $$

3 $$ \mathrm{M}={\mathrm{i}}_3+\mathrm{aX}+{\mathrm{e}}_3 $$

where *Y* is the number of physical unhealthy days (dependent variable), *X* is the obesity status (independent variable), *M* is the inflammation status (mediating variable), *c* is the coefficient relating X to Y, *c’* is the coefficient relating X to Y adjusted for M, *b* is the coefficient relating M to Y adjusted for X, *a* is the coefficient relating X to M, *i*_*1*_*-i*_*3*_ are intercepts and *e*_*1*_*-e*_*3*_ are residuals, respectively.

## Results

### Participants

The general characteristics of the survey respondents are listed in Table 1, with continuous measures reported as the mean (+ standard deviation), and categorical measures expressed as frequencies. The average age of the sampled population was 51.3 years (+ 17.85 years), with approximately 49% male subjects. The proportions of normal weight, overweight and obese subjects were 27, 35 and 38%, respectively. CRP levels were in the ‘normal’ range for 51% of the subjects, ‘elevated’ in 37%, and ‘clinically raised’ in 11% of subjects. With the exception of age, sex and race, the data displayed varying levels of missingness for all other variables.

**Table 1:** Demographic and Medical Characteristics of Study Subjects

Variable	Mean (SD) or frequency (%)	% missing
Sample size = 6325		
Age (yrs.)	51.30(17.85)	0
Male	48.96%	0
Race		
-*Mexican American (1)*	17.28%	0
*-Other Hispanic (2)*	11.38%	0
*-Non-Hispanic White (3)*	46.75%	0
*-Non-Hispanic Black (4)*	20.63%	0
*-Other Race (Multiracial) (5)*	4.03%	0
HSQ470 (days)	4.49(8.71)	11.74
HSQ480 (days)	4.09(8.28)	11.71
BMI (kg/m^2^)	29.34(6.77)	5.66
*-normal weight(18.5–24.99)*	*27.1%*	
*-overweight(25–29.99)*	*34.8%*	
*-obese(≥ 30)*	*38.0%*	
CRP(mg/dl)	0.46(0.89)	9.92
*-non-elevated (< 0.22)*	*51.3%*	
*-elevated (≥ 0.22- < 1.0)*	*37.4%*	
*-clinically raised (≥ 1.0)*	*11.3%*	
SMQ040 (=1) [smoking]	21.97%	52.29
MCQ010 (=1) [asthma]	14.01%	0.09
MCQ220 (=1) [cancer]	10.12%	0.17
MCQ160A (=1) [arthritis]	32.21%	0.16
Any heart disease (=1)	9.38%	0.01
Anti-inflammatory drug use	13.72%	38.02

Data is presented as mean(SD) for continuous variables and as frequency(%) for categorical variables. The percent of data missing for each variable is indicated. Inflammation-related variables are coded (as per NHANES 2005–2008) as follows: SMQ040 (current smoking status), MCQ010 (medical diagnosis of asthma), MCQ220 (medical diagnosis of cancer), MCQ160A (medical diagnosis of arthritis), Any heart disease (medical diagnosis of one or more of heart attack, congestive heart failure or coronary heart disease)

### Relationship between body mass index and C-reactive protein

Quantile-quantile plots demonstrated that CRP and BMI values were better approximated to the normal distribution after log transformation (data not shown). We carried out linear regression to determine the association between CRP levels and BMI. Taking log CRP as the dependent variable and log BMI as the predictor variable, the regression coefficient of BMI was 2.69 (95% CI: 2.50, 2.88) (Additional file 1: Table S1, Figure S1, Text 3 and 4), indicating a statistically significant association between BMI and CRP in the study population.

### Relationship of body mass index (BMI) and C-reactive protein (CRP) to physical and mental healthy days

Considering physical unhealthy days (HSQ470) as a binary response, we performed logistic regression with BMI groups (normal, overweight and obese) (normal group as reference) (Table 2, model 1). The estimated odds ratio for overweight subjects was 1.06 (95% CI: 0.76, 1.47) and that for obese subjects was 1.59 (95% CI: 1.15, 2.21). Thus, compared to a normal-weight person, an overweight person (25 ≤ BMI ≤ 29.9) was 1.06 times more likely, and an obese person (BMI ≥ 30) 1.59 times more likely to experience > 15 physical unhealthy days in a month. Only the estimated OR for the obese, but not overweight, individuals were statistically significant (95% CI excluded 1). In contrast, neither overweight nor obese individuals were significantly associated to mental unhealthy days (95% CI includes 1) (Table 2, model 2).

**Table 2:** Relationship of Physical and Mental Healthy Days to BMI and CRP levels

Model	Dependent Variable	Parameter	OR (95% CI)
Model 1	HSQ470 (physical)	(Intercept)	0.06 (0.04,0.09)
		overweight	1.06 (0.76,1.47)
		obese	1.59 (1.15,2.21)
Model 2	HSQ480 (mental)	(Intercept)	0.06 (0.05,0.09)
		overweight	1.20 (0.86,1.68)
		obese	1.25 (0.89,1.75)
Model 3	HSQ470 (physical)	(Intercept)	0.06 (0.04,0.07)
		elevated CRP	1.61 (1.23,2.12)
		clinically raised CRP	2.45 (1.84,3.26)
Model 4	HSQ480 (mental)	(Intercept)	0.07 (0.05,0.09)
		elevated CRP	1.05 (0.79,1.40)
		clinically raised CRP	1.66 (1.26,2.19)
Model 5	HSQ470 (physical)	(Intercept)	0.05 (0.04,0.07)
		overweight	0.98 (0.70,1.35)
		obese	1.26 (0.92,1.74)
		elevated CRP	1.51 (1.14,2.0)
		clinically raised CRP	2.21(1.55,3.16)

Results include estimates of odds ratio (OR) and corresponding 95% confidence intervals under different models indexed by varying dependent variables. The OR is interpreted as the relative changes in odds for physical (HSQ470 > 15 days) or mental (HSQ480 > 15 days) unhealthy days upon changes in the categories of the explanatory variables (BMI and/or CRP)

Data was analyzed using sampling weighted generalized linear models (logistic) as described under Methods. Model specifications are as follows: Model 1, HSQ470 vs. BMI; Model 2, HSQ480 vs. BMI; Model 3, HSQ470 vs. CRP; Model 4, HSQ480 vs. CRP; Model 5, HSQ470 vs. BMI and CRP

To further assess the relationship between the obesity class and physical/mental unhealthy days, we focused only on obese subjects (BMI ≥ 30), divided into 5 subclasses according to increasing values of BMI (Additional file 1: Table S2 and S3). Subjects in the two highest classes of obesity (class IV, BMI 50.0–59.9 and class V, BMI ≥ 60) were found to be significantly associated to physical unhealthy days, compared to baseline (class I obesity, BMI 30.0–34.9). Only class V obesity subgroup was found to be significantly associated to HSQ480, with higher BMI associated with a reduced probability for mental unhealthy days. Although this finding is counterintuitive, we note that the statistical estimates may be unstable due to the very low subject numbers in this group (15 individuals, < 1% of total BMI ≥ 30 population).

Next, we assessed the relationship between plasma CRP levels (with non-elevated CRP class as reference) and the number of physical unhealthy days (Table 2, model 3). The estimated OR of elevated CRP was 1.61 (95% CI: 1.23, 2.12) and that of clinically raised CRP was 2.45 (95% CI: 1.84, 3.26), suggesting statistically significant associations for both CRP categories. The association between elevated CRP to mental unhealthy days (HSQ480) was not significant (OR = 1.05, 95% CI: 0.79, 1.40); however, clinically raised CRP was significantly associated to mental unhealthy days, (OR = 1.66, 95% CI: 1.26, 2.19) **(**Table 2, model 4).

We next modeled both BMI groups and CRP categories as explanatory variables to ascertain their relative contribution to physical unhealthy days. The estimated odds ratios were 0.98 and 1.26 for overweight and obese BMI groups, respectively, and, 1.51 and 2.21 for elevated CRP and clinically raised CRP, respectively (Table 2, model 5). However, both the 95% CIs corresponding to the overweight (95% CI: 0.70, 1.35) and obese group (95% CI: 0.92, 1.74) now included 1, whereas the corresponding CIs for elevated CRP (95% CI: 1.14, 2.00) and clinically raised CRP (95% CI: 1.55, 3.16) excluded 1. In other words, when both CRP and BMI are included as explanatory variables in the same model, the significant associations observed earlier between BMI level and physical unhealthy days was no longer present. In the context of a mediation-framework according to Eqs. 1–3 listed under Methods, we observed significant relations for the coefficients *a*, *b* and *c* but not for *c’*, suggesting that inflammation may function as a possible mediator of the observed association between obesity and physical unhealthy days (Fig. 1).

### Effect modification analysis:

We carried out an effect modification analysis on the relationship of HSQ470 to BMI and CRP by including gender, age-class and race in the models. The interaction effects between `overweight and gender’, `obese and gender’ and ‘overweight and Race-5’ were significant (Table 3). For example, within the overweight category, a male was 0.42 times less likely to experience > 15 physical unhealthy days compared to a female. All other interactions were non-significant. For HSQ480 (mental unhealthy days), we observed significant interaction effects due to ‘obese and gender’; ‘clinically raised CRP and gender’; ‘overweight and Age (45-65yrs)’; ‘obese and Age (45-65yrs)’; ‘clinically raised CRP and Age (45-65yrs)’; ‘clinically raised CRP and Age (>65yrs)’, and, ‘overweight and Race-5′ (Additional file 1: Table S4). All other interactions effects were non-significant. These results suggest that the observed association between adiposity or CRP and physical/mental healthy days are modifiable to some extent by age and gender. However, the apparent modification of the association between overweight and HSQ470/ HSQ480 by Race-5 has to be interpreted with caution due to the very low numbers of subjects belonging to this category (< 5% of the surveyed population, Table 1).

**Table 3:** Effect modification for outcome variable HSQ470

	Outcome Variable HSQ470		
	OR (95% CI)		OR (95% CI)
Effect modification due to GENDER			
(Intercept)	0.06 (0.04, 0.09)	(Intercept)	0.06 (0.04, 0.09)
Overweight	1.60 (1.01, 2.54)	elevated CRP	1.72 (1.18, 2.51)
Obese	1.96 (1.24, 3.11)	clinically raised CRP	2.38 (1.61, 3.52)
GENDER1	1.26 (0.80, 1.99)	GENDER1	0.92 (0.63, 1.33)
Overweight:GENDER1	*0.42 (0.26, 0.68)*	elevated CRP: GENDER1	0.83 (0.57, 1.20)
Obese:GENDER1	*0.62 (0.39, 0.99)*	clinically raised CRP: GENDER1	1.03 (0.70, 1.51)
Effect modification due to AGE			
(Intercept)	0.03 (0.02, 0.06)	(Intercept)	0.03 (0.02, 0.05)
Overweight	1.16 (0.64, 2.12)	elevated CRP	1.72 (1.03, 2.89)
Obese	1.64 (0.90, 3.01)	clinically raised CRP	2.16 (1.28, 3.63)
AGE (45-65 yrs)	2.68 (1.47, 4.89)	AGE (45-65 yrs)	2.67 (1.60, 4.45)
AGE (>65 yrs)	3.40 (1.86, 6.21)	AGE (>65 yrs)	2.53 (1.51, 4.24)
Overweight:AGE (45-65 yrs)	0.79 (0.43, 1.45)	elevated CRP: AGE (45-65 yrs)	0.79 (0.47, 1.33)
Obese:AGE (45-65 yrs)	0.89 (0.49, 1.63)	clinically raised CRP: AGE (45-65 yrs)	0.89 (0.53, 1.51)
Overweight:AGE (>65 yrs)	0.78 (0.43, 1.44)	elevated CRP: AGE (>65 yrs)	1.01 (0.60, 1.70)
Obese:AGE (>65 yrs)	0.82 (0.45, 1.50)	clinically raised CRP: AGE (>65 yrs)	1.57 (0.93, 2.66)
Effect modification due to Race			
(Intercept)	0.05 (0.02, 0.11)	(Intercept)	0.05 (0.03, 0.09)
Overweight	1.05 (0.48, 2.30)	elevated CRP	1.22 (0.66, 2.24)
Obese	1.42 (0.65, 3.12)	clinically raised CRP	2.23 (1.23, 4.04)
Race2	1.22 (0.56, 2.67)	Race2	1.40 (0.77, 2.54)
Race3	1.32 (0.60, 2.88)	Race3	1.20 (0.66, 2.17)
Race4	1.05 (0.48, 2.31)	Race4	1.20 (0.66, 2.19)
Race5	0.92 (0.42, 2.02)	Race5	0.63 (0.33, 1.22)
Overweight:Race2	1.53 (0.70, 3.37)	elevated CRP:Race2	1.00 (0.54, 1.85)
Obese:Race2	0.97 (0.44, 2.14)	clinically raised CRP:Race2	1.15 (0.60, 2.21)
Overweight:Race3	1.02 (0.46, 2.24)	elevated CRP:Race3	1.47 (0.79, 2.73)
Obese:Race3	1.17 (0.54, 2.58)	clinically raised CRP:Race3	1.20 (0.65, 2.19)
Overweight:Race4	0.95 (0.43, 2.09)	elevated CRP:Race4	0.96 (0.50, 1.83)
Obese:Race4	1.09 (0.50, 2.40)	clinically raised CRP:Race4	0.63 (0.35, 1.15)
Overweight:Race5	*0.30 (0.14, 0.67)*	elevated CRP:Race5	0.91 (0.33, 2.51)
Obese:Race5	0.58 (0.26, 1.29)	clinically raised CRP:Race5	1.11 (0.37, 3.37)

The modification of the association between physical healthy days (HSQ470) and BMI or CRP was investigated. Data was analyzed using sampling weighted generalized linear models (logistic) as described under Methods.

Significant associations are shown in italics

### Sensitivity analysis

We performed sensitivity analysis on the relationship of HSQ470 to BMI groups and CRP classes by varying the cut-off value for HSQ470 = 1 from 15 to 12, 13, 14, 16, 17 and 18 days. The obese class was significantly associated to HSQ470 for all the cut-off values tested with stable odds ratio estimates (Table 4). On the other hand, the odds-ratios for overweight were non-significant for all HSQ470 cut-off values tested, consistent with the original findings. Similarly, the odds-ratios corresponding to the different CRP classes (elevated and clinically raised CRP) with different HSQ470 cut-off values were significant, agreeing again with the primary results (HSQ470 cut-off value = 15). These results suggest that the identified associations between BMI or CRP and HRQOL are robust to the threshold used for defining physical unhealthy days.

**Table 4:** Sensitivity Analysis with respect to different cut-off values of HSQ470 vs BMI and CRP

	OR (95% CI) (outcome variable HSQ470 vs BMI Class)					
Cut-off of HSQ470	12	13	14	16	17	18
Intercept	0.08 (0.06,0.11)	0.08 (0.06,0.11)	0.07 (0.05,0.1)	0.06 (0.04,0.08)	0.06 (0.04,0.08)	0.06 (0.04,0.08)
Overweight	1.21 (0.91,1.59)	1.19 (0.9,1.58)	1.2 (0.89,1.61)	1.03 (0.74,1.43)	1.02 (0.73,1.42)	1.02 (0.73,1.42)
Obese	1.69 (1.27,2.23)	1.7 (1.28,2.25)	1.66 (1.23,2.23)	1.6 (1.15,2.23)	1.58 (1.13,2.2)	1.54 (1.1,2.15)
	OR (95% CI) (outcome variable HSQ470 vs CRP Class)					
Cut-off of HSQ470	12	13	14	16	17	18
Intercept	0.08 (0.06,0.1)	0.08 (0.06,0.1)	0.07 (0.05,0.09)	0.05 (0.04,0.07)	0.05 (0.04,0.07)	0.05 (0.04,0.07)
elevated CRP	1.54 (1.22,1.96)	1.53 (1.2,1.93)	1.55 (1.21,1.98)	1.7 (1.29,2.24)	1.7 (1.28,2.24)	1.73 (1.3,2.28)
clinically raised CRP	2.34 (1.83,2.99)	2.36 (1.84,3.03)	2.46 (1.89,3.2)	2.59 (1.94,3.45)	2.64 (1.97,3.54)	2.7 (2.01,3.62)

The threshold for physical unhealthy days was varied from 12 to 18 days and the effects on the association to BMI or CRP classes was evaluated (upper and lower panels of table, respectively). Data was analyzed by sampling weighted generalized linear models as described under Methods

### Relationship of CRP to physical and mental unhealthy days after adjustment for other sources of inflammation

We next investigated whether the effects of CRP classes on physical unhealthy days could be confounded by some of the more common sources of inflammation encountered in the study population (mediator outcome confounding). We carried out multivariable logistic regression analysis by including demographics (age, gender), pro-inflammatory medical conditions, use of common anti-inflammatory/pain medications, and current smoking status, in addition to CRP and BMI categories in the model (Table 5**)**. The CRP.Class variable remained significantly associated to physical unhealthy days, for both the elevated CRP (OR = 1.34, 95% CI: 1.00, 1.79) and clinically raised CRP (OR = 1.71, 95% CI:1.18, 2.48), even after adjustment. A similar analysis against mental unhealthy days showed the association of CRP classes and BMI groups to be non-significant, although significant associations were observed for presence of asthma, presence of arthritis, current smoking status, occurrence of any heart disease and gender **(**Additional file 1: Table S5).

**Table 5:** Multivariable logistic regression analysis of the association of CRP to physical unhealthy days

Parameter	OR (95% CI)	*p*-value
(Intercept)	0.04 (0.02, 0.06)	< 0.01
Overweight	0.95 (0.68, 1.33)	0.76
Obese	1.12 (0.79, 1.58)	0.52
CRP.class (2)	1.34 (1.00, 1.79)	0.05
CRP.class (3)	1.71 (1.18, 2.48)	0.01
Anti-inflammatory Drug Use (1)	2.40 (1.74, 3.3)	< 0.01
AGEclass (2)	1.88 (1.35, 2.63)	< 0.01
AGEclass (3)	1.79 (1.2, 2.68)	< 0.01
MCQ010 (1)	1.36 (1.01, 1.83)	0.05
MCQ220 (1)	1.36 (0.96, 1.95)	0.09
MCQ160A (1)	2.32 (1.72, 3.13)	< 0.01
GENDER (1)	0.88 (0.68, 1.15)	0.35
SMQ040 (1)	0.83 (0.69, 1.00)	0.05
Any Heart Disease (1)	1.63 (1.14, 2.32)	0.01

Results include estimates of odds ratio (OR) and corresponding 95% confidence intervals. The OR is interpreted as the increase in odds for physical (HSQ470 > 15 days) unhealthy days upon changes in the categories of the explanatory variables. Data was analyzed using sampling weighted generalized linear models (logistic) as described under Methods

## Discussion

The present study was undertaken to better define the relationship between obesity, systemic inflammation and measures of HRQOL. We used data from a US population based survey (NHANES 2005–2008) to estimate effects of increasing body mass and increasing inflammation on the number of physical and mental unhealthy days reported by participants. We also tested the impact of common inflammation regulators (inflammatory disease, anti-inflammatory drug use, and smoking) on the association between the inflammation marker CRP, and HRQOL (based on the CDC HRQOL-4 questionnaire). Compared to the more detailed Medical Outcomes Study Short Form 36 (SF-36), the CDC’s “healthy days” serves as a simple proxy measure of HRQOL. It measures perceptions of physical and mental health using one question each, eliminating the need for complex weighting factors to calculate summary scores.

In previous studies, Hassan et al. [32] assessed a US-based sample with the CDC-HRQOL-4 and reported greater likelihood of poor physical and mental quality of life in participants with obesity. Renzaho et al. [34] sampled an Australian population with SF-36 and found that physical, but not mental, HRQOL scores were negatively associated with BMI. Serrano-Aguilar et al. [35] analyzed a European sample using the EuroQol-5d assessment and found that participants with BMI > 40 had lower HRQOL scores than normal weight participants. These findings agree with the positive association between BMI and number of physical unhealthy days observed in the current study in unadjusted models, and also support the general lack of association between BMI and mental unhealthy days [34].

We next conducted an exploratory analysis to examine whether obesity-associated systemic inflammation could potentially mediate some of the observed association between obesity and HRQOL (physical or mental unhealthy days), by employing the well-known causal steps approach to mediation by Barron and Kenny [68, 69]. This approach consists of 4 steps to establish mediation, namely (i) a significant relation of the independent variable (obesity) to the dependent variable (number of physical unhealthy days) (Table 2, model 1); (ii) a significant relation of the independent variable to the hypothesized mediating variable (systemic inflammation) (Additional file 1**:** Table S1); (iii) a significant relation of the mediating variable to the dependent variable in the presence of independent variable (Table 2, model 5); (iv) a smaller absolute coefficient relating the independent variable to the dependent variable in the presence of the mediating variable (Table 2, model 5). All four conditions were satisfied in the analysis of systemic inflammation as a possible mediator of the relation between obesity and physical healthy days. Notably, the association between BMI and physical HRQOL was non-significant after systemic inflammation (CRP levels) was included in the regression model **(**Table 2, model 5).

We further investigated whether systemic inflammation was itself a predictor of HRQOL in the NHANES cohort, and whether the association was modified in the presence of other factors such as obesity, common inflammatory disease, anti-inflammatory drug use, etc. We based our analysis on the rationale that chronic inflammation is an important index of population health and provides a unifying pathological mechanism for many seemingly unrelated diseases [70]. Recent research focusing on general associations between specific inflammatory chronic conditions (e.g. asthma, irritable bowel syndrome, Crohn’s disease, chronic prostatitis, etc.) and health outcomes have also reported significant associations between symptom severity and HRQOL reductions [71–76]. These reductions are further compounded by psychological distress [77] and allostatic load [78]. Literature on inflammatory disease and HRQOL suggests that these negative associations may be partly mediated by the common medical consequences of chronic illness [79]. For example, pain and disability linked to chronic inflammation has been found to play a small but significant mediating role in the overall HRQOL reduction in older adults [80].

In our analysis, systemic CRP levels were positively and significantly associated with the number of physical and mental unhealthy days, even after adjustments for sex, age, pro-inflammatory co-morbidities, and anti-inflammatory/analgesic drug use. Importantly, the observed association to physical unhealthy days persisted even for levels of inflammation below the clinical threshold. Visser et al. [44] introduced the classification scheme of sub-clinical ‘elevated CRP (≥0.22 mg/dl)’ and ‘clinically raised CRP (≥1.0 mg/dl)’ and identified an association of the former with overweight and obesity. In other studies, sub-clinical CRP has been associated with increased risk of cardiovascular disease-related mortality in healthy subjects [81]. Our study now further demonstrates the importance of sub-clinical CRP levels in the domain of HRQOL.

We now discuss some potential limitations of the study and differences from previously published reports. While our study used the number of healthy days as the HRQOL metric, previous studies utilized composite HRQOL measures, based on a summation over several sub-domain scores. Additionally, differences in the sampled populations between the studies could also potentially influence the current findings. Also, since the assessment by CDC HRQOL-4 is based on self-reporting, the study results are also potentially susceptible to the risk of recall error and misreport. In this study, we applied the multiple imputation method to address the missing data, based on the underlying assumption of ‘missing at random’(MAR) [82], wherein the systematic differences between the missing and observed values are entirely explicable by other observed variables. Under MAR, the multiple imputation approach maintains the benefits of maximum likelihood estimation, while also allowing for uncertainty due to imputation (infeasible under single imputation) to be included during data analysis. In our study, we have assumed MAR for all the variables, including smoking and anti-inflammatory drug uses that have high proportion of missing values (52 and 38%, respectively). Since the study sample size is reasonably large (6325), we expect the missing values imputation to remain robust, even for variables with a high proportion of missing values. Another possible limitation of the current work is that the mediation analysis has been conducted on a cross-sectional design (the only design available for this type of study at the moment). Although several studies in the mediation literature have employed cross-sectional designs, these designs lack the ability to formally support causal inference and instead must depend on a priori assumptions, based on strong biological rationale, to infer mediation [83]. As explained by Maxwell and Cole [49], cross-sectional approaches to mediation typically generate biased estimates in the absence of true time precedence data [84]. In our case, several biological experiments, including Mendelian randomization studies, strongly implicate the precedence of obesity over chronic inflammation reflected in rising CRP levels [23]. Also as discussed earlier [25–29], other studies have shown obesity-associated inflammation to be causally linked to various biologic endpoints. Finally, the ability of disease-associated inflammation to alter HQOL has also been recently demonstrated for depression and schizophrenia [41, 42]. Given these observations, we think there are reasonable biological grounds for exploring a possible mediation-framework in our study. We would, however, caution about the exploratory nature of the current analysis and emphasize it as only hypothesis-generating at present.

## Conclusion

In conclusion, a population-based analysis investigating the roles of obesity and systemic inflammation on indices of health-related quality of life suggests inflammation as a possible mediator of the negative associations between body mass index and the number of reported physical healthy days. Sub-clinical inflammation also appears to be an independent predictor of physical and mental healthy days in the general population. In light of these observations, the relationship of systemic inflammation to quality of life need to be further investigated, and a distinction made between clinically-raised high CRP levels and lower (but possibly chronic) elevations in CRP that can still significantly affect health related quality of life.

## Additional file

Additional file 1: Exploring the associations between systemic inflammation, obesity and healthy days: a health related quality of life (HRQOL) analysis of NHANES 2005–2008. (DOCX 158 kb)

## Abbreviations

BMI: Body Mass Index
CRP: C-reactive protein
HRQOL: Health Related Quality of Life
HSQ470: Health Status Questionnaire 470
HSQ480: Health Status Questionnaire480
NHANES: National Health and Nutrition Examination Survey
