# Supplementary material for: Exploring the associations between systemic inflammation, obesity and healthy days: a health related quality of life (HRQOL) analysis of NHANES 2005–2008
Source: BMC Obes. 2018 Aug 6;5:21. doi: 10.1186/s40608-018-0196-2 (PMC6091152; doi:10.1186/s40608-018-0196-2)
Supplement: Supplementary file 1 — Exploring the associations between systemic inflammation, obesity and healthy days: a health related quality of life (HRQOL) analysis of NHANES 2005–2008. (DOCX 158 kb) [file 40608_2018_196_MOESM1_ESM.docx]

**Additional file: Association of Systemic Inflammation and Obesity to Physical and Mental Healthy Days: analysis of an US population-based survey (NHANES 2005-2008)**

**Supplementary Text 1: Calculation of sample weights**

The sampling weights are created in NHANES to address the complexity of the survey design such as oversampling, survey non-response, and post-stratification in a multistage survey. An analysis of NHANES data, using the sampling weights, enables us to represent the results that are valid for US civilian Census population. As mentioned in <https://www.cdc.gov/nchs/tutorials/nhanes/SurveyDesign/Weighting/OverviewKey.htm>. , in the NHANES, a sample weight is created in three steps: I) Calculation of base weight, II) adjustment for non-response, and III) post-stratification adjustment to match 2000 US Census population totals. In the first step, the base weight is defined as,

$Base Weights=\frac{1}{Final probability}$*, where*

*Final probability = probability of the PSU being selected*

*X probability of a segment of the PSU being selected*

*X probability of a household being selected*

*X probability of an individual being selected,*

Where, PSU is primary sampling units. In the second step, base weights are adjusted for non-response to the in-home interview and/or mobile exam center (MEC). An individual is a non-responder to the interview if s/he is selected to be in the sample but did not participate in the in-home interview. On the other hand, if an individual who agreed to complete the interview but do not agreed to come for MEC portion of the survey. In the third step, the weights are post-stratified to match the population totals for each sampling subdomain (for details see: <https://www.cdc.gov/nchs/tutorials/nhanes/SurveyDesign/Weighting/OverviewKey.htm>). After all the adjustments mentioned above, the sampling weights can be used in the statistical analysis to calculate the estimates that represent the US civilian noninstitutionalized populations.

**Supplementary Text 2:**

**BRFSS - Healthy Days Core Module (CDC HRQOL-4)**

The CDC HRQOL questions have undergone cognitive testing and demonstrated criterion validity with the Short-Form 36, content and construct validity, predictive validity, internal consistency, test-retest reliability, and measurement invariance in persons with and without disability. The HRQOL measures have been mapped or cross-walked to other scales and can be used to estimate health-preference scores for cost-utility analyses. Validation studies can be accessed at: http://www.cdc.gov/hrqol/publications_topic.htm.

In 2001, a review of quality of life (QOL) indices identified 14 criteria for determining the validity and usefulness of QOL indices for public policy (Hagerty et al 2001). The review rated the CDC HRQOL index favorably as a measure of QOL for the health domain (Hagerty et al 2001).

**References:**

Hagerty, M.R., Cummins, R.A., Ferriss, A.L., Land, K., Michalos, A.C., Peterson, M., Sharpe, A., Sirgy, J., & Vogel, J. (2001). Quality of life indexes for national policy: review and agenda for research. Social Indicators Research, 55, 1-96.

- The CDC Healthy Days measures have been a part of the core BRFSS survey (asked of all respondents) since 1993.
- Access to data: BRFSS Survey Data (Year Index)

**Questions:**

1. Would you say that in general your health is excellent, very good, good, fair, or poor?
2. Now thinking about your physical health, which includes physical illness and injury, for how many days during the past 30 days was your physical health not good?
3. Now thinking about your mental health, which includes stress, depression, and problems with emotions, for how many days during the past 30 days was your mental health not good?
4. During the past 30 days, for about how many days did poor physical or mental health keep you from doing your usual activities, such as self-care, work, or recreation?

**Supplementary Table 1: Linear Regression of log (CRP) on log (BMI) values from the NHANES 2005-2008 study sample.** Results include estimates of the regression coefficients, their standard errors and corresponding 95% confidence intervals. The regression coefficient is an estimate of the change in log CRP concentration per unit change in log BMI.

| **Parameter** | **Estimate (95% CI)** |
| --- | --- |
| Intercept | -10.56 (-10.77, -10.34) |
| log(BMI) | 2.69 ( 2.50, 2.88 ) |

**Supplementary Text 3:**

**Residual Analysis:** The residual plots (**Supplementary Figure 1**) corresponding to the regression model with the dependent variable log CRP and the predictor variable log BMI from all the five imputed data sets. The residual plots are showing that residuals (vertical axis in the plots) are randomly distributed around zero.

**Supplementary Figure 1: Residual Plots from 5 imputed data sets (row wise 1 to 5)**

| **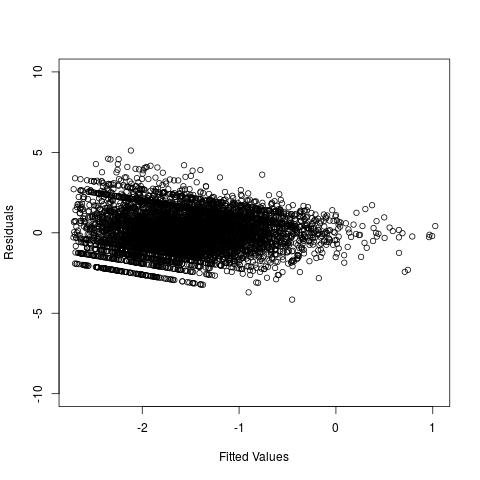** | **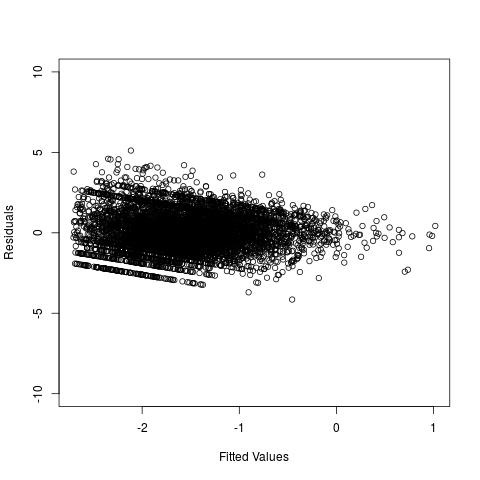** |
| --- | --- |
| **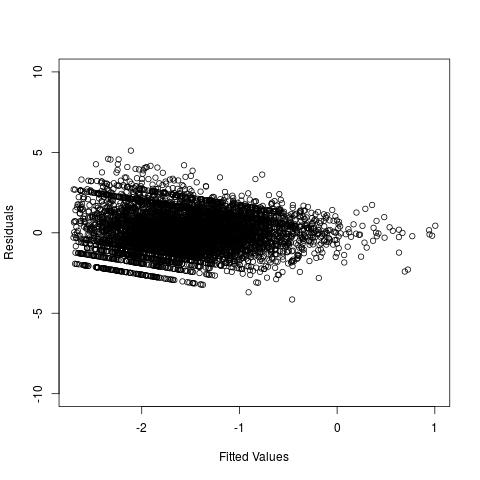** | **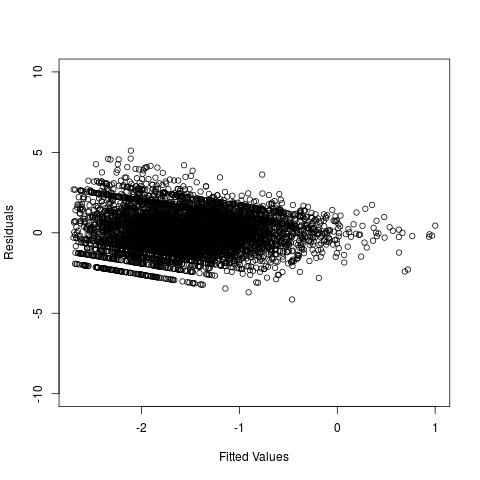** |
| **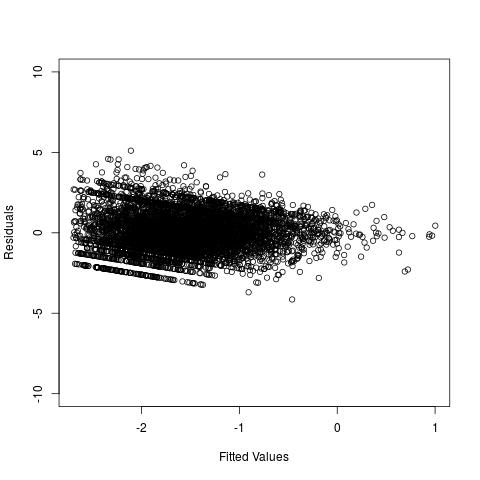** |  |

**Supplementary Text 4:**

**R^2^ Values:** The values of the R^2^ from the five imputed data sets are 0.181, 0.181, 0.178, 0.178, 0.178. We can say that the average R^2^ value is 0.18 based on the 5 imputed data sets [1]. Note that, in the model, we have used log CRP as the dependent variable and log BMI as the predictor variable. In a simple linear model (without any weight) with single predictor, the R^2^ directly reflects the strength of the correlation. However, when weight is used to adjust the selection bias, as done in the manuscript to incorporate survey weights, the R^2^ may not reflect the correlation of interest.

Supplementary Table 2: Relationship of HSQ470 on the extreme obesity classes when the baseline is class I obesity (BMI 30.0-34.9). Data was analyzed using sampling weighted generalized linear models (logistic) as described under Methods.

|  | OR (95% CI) |
| --- | --- |
| (Intercept) | 0.09 (0.06, 0.13) |
| Class II Obesity (BMI 35.0-39.9) | 1.32 (0.87, 2.01) |
| Class III Obesity (BMI 40.0-49.9) | 1.23 (0.81, 1.85) |
| Class IV Obesity (BMI 50.0-59.9) | 2.02 (1.35, 3.03) |
| Class V Obesity (BMI >60.0) | 1.53 (1.02, 2.29) |

Supplementary Table 3: Relationship of HSQ480 on the extreme obesity classes when the baseline is class I obesity (BMI 30.0-34.9). Data was analyzed using sampling weighted generalized linear models (logistic) as described under Methods.

|  | OR (95% CI) |
| --- | --- |
| (Intercept) | 0.08 (0.05, 0.12) |
| Class II Obesity (BMI 35.0-39.9) | 1.06 (0.69, 1.63) |
| Class III Obesity (BMI 40.0-49.9) | 1.01 (0.66, 1.56) |
| Class IV Obesity (BMI 50.0-59.9) | 0.66 (0.43, 1.02) |
| Class V Obesity (BMI >60.0) | 0.000002 (0.0000013, 0.0000031) |

**Supplementary Table 4: Effect modification for outcome variable HSQ480.** Data was analyzed using sampling weighted generalized linear models (logistic) as described under Methods.

|  | **Outcome Variable HSQ480** | | |
| --- | --- | --- | --- |
|  | OR (95% CI) |  | OR (95% CI) |
| **Effect modification due to GENDER** | | | |
| (Intercept) | 0.07 (0.04,0.1) | (Intercept) | 0.09 (0.06, 0.12) |
| Overweight | 1.46 (0.93,2.29) | CRP.class2 | 0.97 (0.67, 1.39) |
| Obese | 1.54 (0.98,2.42) | CRP.class3 | 1.33 (0.93, 1.91) |
| GENDER1 | 0.91 (0.58,1.43) | GENDER1 | 0.63 (0.44, 0.89) |
| Overweight:GENDER1 | 0.69 (0.44,1.08) | CRP.class2:GENDER1 | 1.11 (0.76, 1.61) |
| Obese:GENDER1 | **0.58 (0.37,0.91)** | CRP.class3:GENDER1 | **1.54 (1.07, 2.24)** |
|  |  |  |  |
| **Effect modification due to AGE** | | | |
| (Intercept) | 0.05 (0.03,0.08) | (Intercept) | 0.06 (0.04, 0.09) |
| Overweight | 1.75 (1.07,2.87) | CRP.class2 | 1.27 (0.83, 1.95) |
| Obese | 1.71 (1.04,2.81) | CRP.class3 | 2.19 (1.45, 3.30) |
| AGEclass2 | 2.09 (1.27,3.42) | AGEclass2 | 1.43 (0.95, 2.14) |
| AGEclass3 | 0.95 (0.58,1.57) | AGEclass3 | 0.95 (0.63, 1.44) |
| Overweight:AGEclass2 | **0.43 (0.26,0.71)** | CRP.class2:AGEclass2 | 0.66 (0.43, 1.01) |
| Obese:AGEclass2 | **0.46 (0.28,0.76)** | CRP.class3:AGEclass2 | **0.64 (0.42, 0.98)** |
| Overweight:AGEclass3 | 0.76 (0.46,1.26) | CRP.class2:AGEclass3 | 0.89 (0.57, 1.37) |
| Obese:AGEclass3 | 0.94 (0.57,1.54) | CRP.class3:AGEclass3 | **0.53 (0.34, 0.82)** |
|  |  |  |  |
| **Effect modification due to Race** | | | |
| (Intercept) | 0.04 (0.02, 0.09) | (Intercept) | 0.05 (0.03, 0.10) |
| Overweight | 1.42 (0.63, 3.21) | CRP.class2 | 0.76 (0.39, 1.50) |
| Obese | 1.30 (0.58, 2.95) | CRP.class3 | 1.98 (1.01, 3.89) |
| Race2 | 3.32 (1.47, 7.50) | Race2 | 1.88 (0.96, 3.68) |
| Race3 | 1.56 (0.69, 3.51) | Race3 | 1.35 (0.69, 2.65) |
| Race4 | 1.92 (0.85, 4.34) | Race4 | 1.73 (0.87, 3.42) |
| Race5 | 1.63 (0.72, 3.68) | Race5 | 1.23 (0.63, 2.40) |
| Overweight:Race2 | 0.57 (0.25, 1.29) | CRP.class2:Race2 | 1.67 (0.85, 3.31) |
| Obese:Race2 | 0.78 (0.34, 1.76) | CRP.class3:Race2 | 1.48 (0.73, 3.00) |
| Overweight:Race3 | 0.94 (0.42, 2.13) | CRP.class2:Race3 | 1.44 (0.73, 2.84) |
| Obese:Race3 | 0.93 (0.41, 2.11) | CRP.class3:Race3 | 0.79 (0.40, 1.55) |
| Overweight:Race4 | 0.69 (0.30, 1.55) | CRP.class2:Race4 | 1.07 (0.51, 2.25) |
| Obese:Race4 | 0.92 (0.41, 2.08) | CRP.class3:Race4 | 0.6 (0.30, 1.21) |
| Overweight:Race5 | **0.23 (0.10, 0.53)** | CRP.class2:Race5 | 1.51 (0.74, 3.08) |
| Obese:Race5 | 1.88 (0.79, 4.47) | CRP.class3:Race5 | 1.10 (0.53, 2.30) |

**Supplementary Table 5. Association of CRP, and other inflammation mediators, on mental unhealthy days (HSQ480) in the NHANES 2005-2008 samples.** Results include estimates of odds ratio (OR) and corresponding 95% confidence intervals. The OR is interpreted as the increase in odds for mental (HSQ480>15 days) unhealthy days upon changes in the categories of the explanatory variables. Data was analyzed using sampling weighted generalized linear models (logistic) as described under Methods.

| **Parameter** | **OR (95% CI)** | **p-value** |
| --- | --- | --- |
| (Intercept) | 0.18 (0.11, 0.30) | <0.01 |
| Overweight | 1.39 (0.97, 1.98) | 0.07 |
| Obese | 1.29 (0.91, 1.84) | 0.15 |
| CRP.class (2) | 0.84 (0.62, 1.14) | 0.27 |
| CRP.class (3) | 1.16 (0.77, 1.74) | 0.49 |
| Anti-inflammatory Drug Use (1) | 1.32 (0.88, 1.97) | 0.19 |
| AGEclass (2) | 1.13 (0.82, 1.57) | 0.45 |
| AGEclass (3) | 0.82 (0.53, 1.26) | 0.36 |
| MCQ010 (1) | 1.62 (1.19, 2.22) | <0.01 |
| MCQ220 (1) | 1.45 (0.94, 2.24) | 0.09 |
| MCQ160A (1) | 1.63 (1.20, 2.20) | <0.01 |
| GENDER (1) | 0.66 (0.51, 0.87) | <0.01 |
| SMQ040 (1) | 0.54 (0.45, 0.65) | <0.01 |
| Any Heart Disease (1) | 1.59 (1.02, 2.48) | 0.04 |

**Supplementary Table 6: Nagelkerke R^2^ and Hosmer Lemeshow test corresponding to Table 5 of the main manuscript:** The Nagelkerke R^2^ values and Hosmer Lemeshow test’s p-values for each of the five imputed datasets. The Nagelkerke R^2^ values are in the range 0.11-0.12 which is quite common in epidemiological studies [2]. A non-significant p-value from Hosmer and Lemeshow test is an evidence of goodness of fit. However, in our case, out of five p-values, two are non-significant and three are significant. Thus, we cannot comment any general statement based on the five imputed datasets with respect to the Hosmer and Lemeshow test.

| Imputed data Set | Nagelkerke R^2^ | Hosmer and Lemeshow test: p-value |
| --- | --- | --- |
| 1 | 0.12 | 0.09 |
| 2 | 0.11 | 0.04 |
| 3 | 0.11 | 0.04 |
| 4 | 0.11 | 0.02 |
| 5 | 0.11 | 0.22 |

References:

1. Sinharay S, Stern HS, Russell D: **The use of multiple imputation for the analysis of missing data**. *Psychol Methods* 2001, **6**(4):317-329.

2. Vostanis P, Graves A, Meltzer H, Goodman R, Jenkins R, Brugha T: **Relationship between parental psychopathology, parenting strategies and child mental health--findings from the GB national study**. *Soc Psychiatry Psychiatr Epidemiol* 2006, **41**(7):509-514.
